# IMB0901 inhibits muscle atrophy induced by cancer cachexia through MSTN signaling pathway

**DOI:** 10.1186/s13395-019-0193-2

**Published:** 2019-03-28

**Authors:** Dong Liu, Xinran Qiao, Zhijuan Ge, Yue Shang, Yi Li, Wendie Wang, Minghua Chen, Shuyi Si, Shu-zhen Chen

**Affiliations:** Institute of Medicinal Biotechnology, Chinese Academy of Medical Sciences & Peking Union Medical College, 1# Tiantan Xili, Dongcheng District, Beijing, 100050 China

**Keywords:** Cancer cachexia, C2C12, C26, Muscle atrophy, Myostatin

## Abstract

**Background:**

Cancer cachexia as a metabolic syndrome can lead to at least 25% of cancer deaths. The inhibition of muscle atrophy is a main strategy to treat cancer cachexia. In this process, myostatin (MSTN) can exert a dual effect on protein metabolism, including inhibition of protein biosynthesis and enhancement of protein degradation. In this study, we will test the effect on muscle atrophy induced by cancer cachexia of IMB0901, a MSTN inhibitor.

**Methods:**

Two high-throughput screening models against MSTN were developed. By screening, IMB0901, 2-((1-(3,4-dichlorophenyl)-1H-pyrazolo [3,4-d] pyrimidin-4-yl) amino) butan-1-ol, was picked out from the compound library. The in vitro cell model and the C26 animal model of muscle atrophy induced by cancer cachexia were used to determine the pharmacological activity of IMB0901. Whether IMB0901 could inhibit the aggravating effect of doxorubicin on muscle wasting was examined in vitro and in vivo.

**Results:**

IMB0901 inhibited the MSTN promoter activity, the MSTN signaling pathway, and the MSTN positive feedback regulation. In atrophied C2C12 myotubes, IMB0901 had a potent efficiency of decreasing MSTN expression and modulating MSTN signaling pathway which was activated by C26-conditioned medium (CM). In C2C12 myotubes, the expressions of three common myotube markers, myosin heavy chain (MyHC), myogenic differentiation 1 (MyoD), and myogenin (MyoG), were downregulated by CM, which could be efficiently reversed by IMB0901 via reduction of ubiquitin-mediated proteolysis and enhancement of AKT/mTOR-mediated protein synthesis. In the C26 animal model, IMB0901 mitigated the weight loss of body, quadricep and liver, and protected the quadriceps cell morphology. Furthermore, IMB0901 decreased the expression of two E3 ligases Atrogin-1 and MuRF-1 in the quadriceps in vivo. At the cellular level, IMB0901 had no influence on anti-tumor effect of three chemotherapeutic agents (cisplatin, doxorubicin, and gemcitabine) and lowered doxorubicin-induced upregulation of MSTN in C2C12 myotubes. IMB0901 did not affect the inhibitory effect of doxorubicin on C26 tumor and delayed the weight loss of muscle and adipose tissue caused by C26 tumor and doxorubicin.

**Conclusions:**

IMB0901 inhibits muscle atrophy induced by cancer cachexia by suppressing ubiquitin-mediated proteolysis and promoting protein synthesis. These findings collectively suggest that IMB0901 is a promising leading compound for the management of muscle atrophy induced by cancer cachexia.

**Electronic supplementary material:**

The online version of this article (10.1186/s13395-019-0193-2) contains supplementary material, which is available to authorized users.

## Background

By definition, cachexia is a severe metabolic syndrome caused by several factors [1, 2] including cancer, sepsis, chronic kidney disease, cardiac failure, and acquired immune deficiency syndrome [3, 4]. Cancer cachexia occurs to over 50% of cancer patients. Unfortunately, it represents poor prognosis and leads to at least 25% of cancer deaths [5]. The most striking feature of cancer cachexia is the uncontrolled and continuous loss of body weight which is mainly reflected by the wasting of adipose tissue and the atrophy of skeletal muscles [6]. Thus, cancer cachexia compromises the life quality of patients and increases the susceptibility to traditional anti-tumor treatment [7].

The skeletal muscle atrophy is a complication secondary to denervation, starvation, aging, disuse, and cachexia [8]. Attenuating muscle atrophy is a main approach to treat cancer cachexia. The molecular mechanism of muscle atrophy is associated with a combination of reduced anabolism and increased catabolism. Smith and Tisdale first discovered the inhibition of protein synthesis induced by cancer cachexia in the mouse model of MAC-16 adenocarcinoma [9]. The excessive protein degradation occurs mainly through the ubiquitin-proteasome and autophagy-lysosome ways [10]. The former is a common manner for protein degradation, in which Atrogin-1 and MuRF-1 as muscle-specific E3 ligases are key proteins mediating in the muscle atrophy of mouse. At the same time, many extracellular ligands (IGF-1, proinflammatory cytokines and the members of TGFβ family etc.) are associated with muscle atrophy induced by cancer cachexia [11–13]. Especially, IGF1-activated PI3K/AKT/mTOR signaling pathway [14] and inflammatory cytokines TNFα, IL6, and IL1α [11] are closely related to muscle atrophy induced by cancer cachexia.

In this pathological process, the members of the TGFβ family, such as myostatin (MSTN) and activin A, can serve a dual role by inhibiting signaling pathway of protein synthesis and activating signaling pathway of protein degradation in cachectic muscle cells [10, 15, 16]. Discovered by McPherron et al. [17] in 1997, MSTN is a negative regulator of skeletal muscle growth and an autocrine or paracrine cytokine predominantly in the type of muscle with highly conserved the nucleotide and amino acid sequences [18].

In the development of cancer cachexia, the appearance of muscle atrophy often predicts high mortality [12]. Inhibition of muscle atrophy is one of the important methods for treating and relieving cancer cachexia. At present, the drugs for treating muscle atrophy induced by cancer cachexia include natural compounds, enzyme inhibitors, beta-adrenergic receptor agonists, and various anti-cytokine drugs [19]. As mentioned above, it is potential for MSTN to be a target of anti-cancer cachexia. Unfortunately, the occurrence, development, and changes of MSTN in the process of cancer have not been fully revealed. Although it is controversial whether MSTN is upregulated in patients or animal models with cancer cachexia, it has been accepted that the inhibition of MSTN or its signaling pathway effectively alleviates muscle atrophy and thus relieves cancer cachexia. Gallot et al. [20] have discovered that the muscle atrophy symptoms have been effectively relieved in the MSTN^−/−^ LLC mice model due to the reduction of the ubiquitin-proteasome and autophagy-lysosome degradations. Zhou et al. [21] have obtained similar results by inhibiting the MSTN signaling pathway. Therefore, we supposed that the development of new MSTN inhibitors have potential application values in the treatment of cancer cachexia [22].

So far, the current MSTN inhibitors are divided into five different types as follows: MSTN antibody, ActRIIB antibody, ActRIIB-Fc, follistatin-AAV gene therapy, and MSTN peptibody in the stage of clinical trial or preclinical study [22]. In terms of chemical structure, most of these inhibitors belong to protein or gene drugs, which limits their extension and application because of the higher expense as compared with small molecule compounds. What is more, there is no MSTN inhibitor developed to target the MSTN expression. It is an urgent need to find new small molecules which inhibits MSTN signaling pathway and/or MSTN expression.

Therefore, a high-throughput drug screening model based on the MSTN promoter activity was established and used as a primary screening. After verification, a high-throughput drug screening based on the MSTN signaling pathway was used as a secondary screening. The candidate compound IMB0901 was selected from 26,000 molecules. The in vitro and in vivo models of cancer cachexia-induced muscle wasting were used to determine the pharmacological activity of IMB0901, and the possible molecule mechanism of IMB0901 was explored. In addition, the effect of IMB0901 on the antitumor effect of chemotherapeutic drugs was examined. It is possible that IMB0901 becomes the leading compound of the MSTN inhibitor.

## Material and methods

### Reagents and antibodies

The cytokine MSTN or its signaling pathway inhibitor SB431542 was purchased from Peprotech or Selleckchem, respectively. Antibodies against Atrogin-1 and MuRF-1 were obtained from ECM Biosciences. Anti-myosin heavy chain was from R&D Systems. Antibodies against Akt, p-Akt (Ser473), p-Akt (Thr308), ubiquitin, p-FOXO3a (Ser 253), and FOXO3a were purchased from Cell Signaling Technology. Anti-MyoG and anti-MSTN antibodies were obtained from Abcam. Anti-MyoD and anti-GAPDH are from Proteintech. The HRP-conjugated anti-mouse and anti-rabbit antibodies were obtained from Zhongshanjinqiao.

### Two stable-transfected cell lines HEK293T-MSTNP and HEK293-SBE

A luciferase reporter vector (pGL4.20, Promega) was used to generate the luciferase recombinant plasmid. The murine MSTN promoter region was acquired by PCR with the forward primer 5′-CCGCTCGAGAATCCCTTGCCTTCATCTG-3′ and the reverse primer 5′-GG AAGATCTTACCGTCCGAGAGACAACC-3′. And then, these PCR products were digested with the restriction enzyme sites *Xhol* I and *Bgl* II. The fragments were inserted into pGL4.20 luciferase reporter vectors to obtain the luciferase recombinant plasmid pGL4.20-MSTNP (Additional file 1: Figure S1). The recombinant plasmid was transfected into HEK293T. The puromycin resistance screening was used to obtain a stable cell line HEK293T-MSTNP.

The pGL4.20 vector was used to generate the luciferase construct pGL4.20-SMAD binding elements (SBE). With transfection and the puromycin resistance screening, the other stable cell line HEK293-SBE was acquired, which was used to screen the compounds with the activity of inhibiting MSTN signaling pathway [23].

### Drug screening by luciferase reporter assays

The stably transfected cells HEK293T-MSTNP (1 × 10^4^) were seeded in 96-well microplates. Incubated with different compounds for 24 h, we measured the luciferase activity using the BrightGlo Luciferase Assay System (Promega) according to the manufacturer’s protocol to evaluate whether this compound can inhibit the MSTN promoter activity.

The possible compounds with potent activity to inhibit MSTN promoter activity were employed to evaluate whether they could inhibit MSTN signaling pathway based on the cell line HEK293-SBE with the method reported by Cash et al. [23]

All the experiments were performed and validated for at least three times.

Then, in similar experimental, we explored the luciferase activities of HEK293T-MSTNP cells that were exposed to MSTN plus IMB0901 or not.

### MTT assay

C2C12 (5 × 10^3^), C26 cells (5 × 10^3^), or HEK293T-MSTNP (1 × 10^4^) were seeded into 96-well plates and after cell adherence was treated with 100 μl of fresh medium containing various concentrations of IMB0901 with or without doxorubicin for 24 h or 48 h. Then, MTT were added into the medium at 37 °C for 4 h. After discarding the medium, the formazan crystals were dissolved with 150 μl DMSO every well and the absorbance at 570 nm was measured with a microplate reader.

### Cell culture, C26-conditioned medium preparation

Murine C2C12 myoblast cell line, purchased from Typical Culture Preservation Commission Cell Band in Shanghai, was maintained in growth medium (GM) containing DMEM supplemented with 10% FBS.

For myogenic differentiation, when the confluence of C2C12 myoblasts reached 80~ 90%, GM was replaced with differentiation medium (DM) consisting of DMEM supplemented with 2% heat-inactivated horse medium (HS) at day 0. DM was renewed every other day. At day 5, the myotubes were fully differentiated.

Murine C26 cell line, obtained from the American Type Culture Collection (ATCC), was grown in RPMI 1640 medium containing 10% FBS. For C26-conditioned medium (CM) collection, C26 cells were seeded in 10-cm culture plates and grown to 80% confluence. After washed with sterile PBS gently, C26 cells in one plate were cultured in 5-ml FBS-free RPMI 1640 medium. For 24-h incubation, the resulting medium was collected and centrifuged at 1200 rpm for 10 min to remove the cell debris. The supernatant was filtered via a 0.2-μm sterile filter. Aliquots of the filtered medium were used immediately or stored at − 80 °C, which was CM used for a cachectic factor.

### C2C12 myotube atrophy model in vitro

CM was diluted 1:5 with DMEM, and 2% HS was also compensated. That is to say, DM containing 1/6 CM was the final factor causing C2C12 myotube atrophy. To determine the efficiency of IMB0901 in CM-induced muscle atrophy in vitro, C2C12 myoblasts (2 × 10^5^) were cultured in six-well plates, and at 80~ 90% cell confluence, we constituted DM containing 1/6 CM plus 0, 2.5, or 5 μM IMB0901 for GM at day 0. The medium was refreshed at day 2 and day 4. At day 5–10, the myotubes were fully differentiated. On this basis, other experiments were performed. There were four groups in this experiment: control (2% HS), CM (2% HS + 1/6 CM), and IMB0901 (2% HS + 1/6 CM + 2.5 or 5 μM IMB0901).

### Animal experiment

All mice that weighed 20~22 g were male and purchased from Beijing HFK Bioscience (Beijing, China), which were allocated randomly into one of three experimental groups: (1) the healthy mice (the control group), (2) the tumor-bearing mice (the C26 group), and (3) the tumor-bearing mice with intraperitoneal injection of IMB0901 in a dose of 30 mg/kg once every other day (the IMB0901 group). For mice of the C26 and IMB0901 groups, C26 tumor slurry was diluted (1:100) and injected into the right flanks of BALB/c mice. The IMB0901 group received intraperitoneal injection starting the second day after tumor implantation. Tumor volumes and animal body weights were measured during the course of the experiment per 2 days. The animals were monitored daily and were euthanized on day 20 following tumor implantation. The carcass weight and the weights of quadriceps, gastrocnemius, WAT (white adipose tissue), and BAT (brown adipose tissue) were immediately harvested and weighed. For subsequent studies, one of two quadriceps from every mouse was fixed in 4% paraformaldehyde, and the other piece was immediately frozen in liquid nitrogen and stored at − 80 °C. The research protocols were consistent with the regulations of Good Laboratory Practice for non-clinical laboratory studies of drugs issued by the National Scientific and Technological Committee of People’s Republic of China.

To determine the influence of IMB0901 on the antitumor effect of doxorubicin, 40 mice were allocated randomly into one of four experimental groups: (1) the healthy mice (the control group), (2) the tumor-bearing mice (the C26 group), (3) the tumor-bearing mice with twice injection of DOX in a dose of 10 mg/kg (the C26 + DOX group), and (4) the tumor-bearing mice with twice injection of DOX in a dose of 10 mg/kg with injection of IMB0901 in a dose of 25 mg/kg once every other day (the C26 + DOX + IMB0901 group). After the mice were euthanized on day 20, the carcass weight and the weights of quadriceps, gastrocnemius, WAT (white adipose tissue), and BAT (brown adipose tissue) were immediately measured.

### Western blotting

The quadriceps of all mice were homogenized, and total protein was extracted from the muscle homogenate or the C2C12 myotubes lysate using RIPA protein lysis buffer with freshly added 1 mM PMSF. The protein concentration was measured by BCA assay. A total of 30 μg protein was subjected to a 10% SDS-PAGE gel to separate the proteins by gel electrophoresis and then transferred onto polyvinylidene fluoride (PVDF) (0.45 μm; Millipore) membranes. The membranes were blocked for 1 h at 37 °C in 5% BSA and incubated with primary antibodies in blocking buffer overnight at 4 °C. The membranes were washed and incubated with the appropriate horseradish peroxidase-conjugated secondary antibody (Invitrogen) in blocking buffer for 2 h at room temperature. Finally, the membranes were washed before detection. Proteins were detected using the electrochemiluminescence (ECL) reagents (Thermo Fisher Scientific) and visualized using a ProteinSimple FluorChem HD2 imaging system. Quantitative analyses of protein expression were performed using Image J software.

### Real-time PCR

Total RNA from C2C12 myotubes was extracted and purified using MiniBEST Universal RNA Extraction Kit (Takara). RNA concentration was quantified by Nanodrop 2000 (Thermo). Complementary DNA was generated by 1 μg RNA using PrimeScript™ II 1st Strand cDNA Synthesis Kit (Takara). Relative expression levels of MSTN were determined with an ABI 7500 Fast Real-Time PCR system. The GAPDH gene was used as an internal reference to normalize target gene expressions. Changes in the mRNA levels of MSTN (the forward primer: ACGTCCAGAGGGATGACAGCAG; the reverse primer: ACATTTGGGCTTGCCATCCGC) related to control were calculated as described previously [24].

### Immunofluorescence

C2C12 myoblasts in 24-well plates were exposed to IMB0901-treated or IMB0901-untreated DM containing 1/6 CM for 5 days. At day 5, C2C12 myotubes were washed with PBS, fixed with 4% paraformaldehyde in PBS for 20 min, and then permeabilized with 0.5% Triton X-100 for 30 min at room temperature. After incubation with the primary anti-MyHC antibody at 4 °C overnight, followed by Alexa Fluor 488-goat anti-mouse IgG antibody (1:1000; Invitrogen) for 1 h at room temperature, the cells were stained by DAPI (10 μg/ml). The cells were imaged using a fluorescence microscope (OLYMPUS, IX73). The number of nuclei per field was quantitated by Image J software.

### Statistics

Data and results were calculated with Excel Software which were expressed as mean ± SD, and then plotted with GraphPad Prism 5. Statistical differences between the two groups were determined by Student’s *t* test. Statistical difference was defined at *P* value < 0.05.

## Results

### Two cell lines HEK293T-MSTNP and HEK293-SBE were obtained

With the construction flowchart in Additional file 1: Figure S1, the recombinant plasmid with luciferase reporter gene based on the MSTN promoter activity was obtained. By the recombinant plasmid transfection and monoclonalization, HEK293T-MSTNP cell line was established. With the similar method, the recombinant plasmid with luciferase reporter gene based on the MSTN signaling pathway and HEK293-SBE cell line were all acquired.

### IMB0901 inhibited the MSTN promoter activity and the MSTN signaling pathway in a dose-dependent manner

**Fig. 1:**
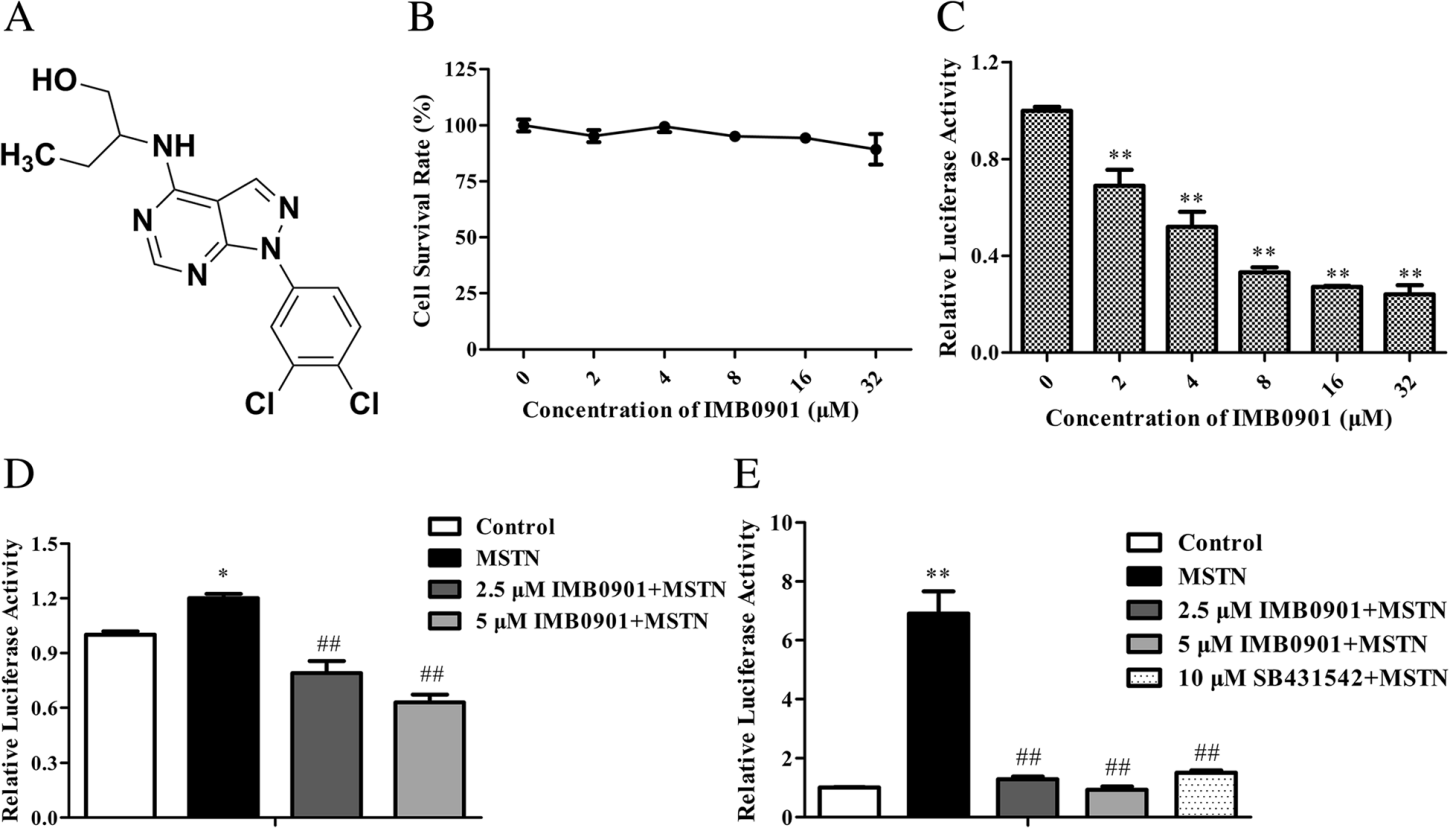
The effect of IMB0901 on MSTN promoter activity and MSTN signaling pathway. **a** The structural formula of IMB0901. **b** The cytotoxicity of IMB0901. MTT assay was used to detect the cell survival rate of HEK293T-MSTNP cells after 24-h treatment with IMB0901 at various concentrations. The values presented means ± SD from three experiments, each performed in triplicate. **c** The inhibition of IMB0901 on the MSTN promoter activity. Bright-Glo™ Luciferase Assay was applied to assess the luciferase activity of HEK293T-MSTNP cells after 24-h direct treatment with IMB0901 at various concentrations. The values were means ± SD from three experiments, each performed in triplicate. ^**^P < 0.01 compared to the control group (0 μM). **d** The inhibition of IMB0901 on the MSTN positive feedback regulation. Bright-Glo™ Luciferase Assay was applied to assess the luciferase activity of MSTN-activated HEK293T-MSTNP cells after 24-h treatment with IMB0901 at various concentrations. The data presented means ± SD from three experiments, each performed in triplicate. *P < 0.05 compared to the control group, ^##^*P* < 0.01 compared to the MSTN group. **e** The inhibition of IMB0901 on the MSTN signaling pathway. Bright-Glo™ Luciferase Assay was used to detect the luciferase activity of MSTN-activated HEK293-SBE cells after 24-h treatment with IMB0901 at various concentrations. The values were means ± SD from three different experiments, each performed in triplicate. ^**^P < 0.01 compared to the control group, ^##^*P* < 0.01 compared to the MSTN group

By screening with HEK293T-MSTNP cells and HEK293-SBE cells, IMB0901 was picked out from 26,000 molecules according to the potency of activity inhibition. IMB0901 was named as 2-((1-(3,4-dichlorophenyl)-1H-pyrazolo [3,4-d] pyrimidin-4-yl) amino) butan-1-ol, with the structural formula shown in Fig. 1a. IMB0901 at less than 32 μM did not inhibit the proliferation of HEK293T-MSTNP cells (Fig. 1b). It was observed that IMB0901 suppressed luciferase activity in a dose-dependent manner (Fig. 1c), and there was a statistical significance between each indicated dose and the control group (*P* < 0.01). On the basis of this model, the data from Fig. 1d demonstrated that MSTN enhanced its promoter activity by itself, which was likewise inhibited by 2.5 or 5 μM IMB0901 (*P* < 0.01 vs. the MSTN group). As shown in Fig. 1e, IMB0901 at a concentration of 2.5 or 5 μM significantly neutralized the MSTN signaling pathway triggered by 10 ng/ml MSTN (*P* < 0.01 vs. the MSTN group), which was similar to 10 μM SB431542.

### IMB0901 downregulated MSTN expression and signaling pathway in atrophied C2C12 myotubes

**Fig. 2:**
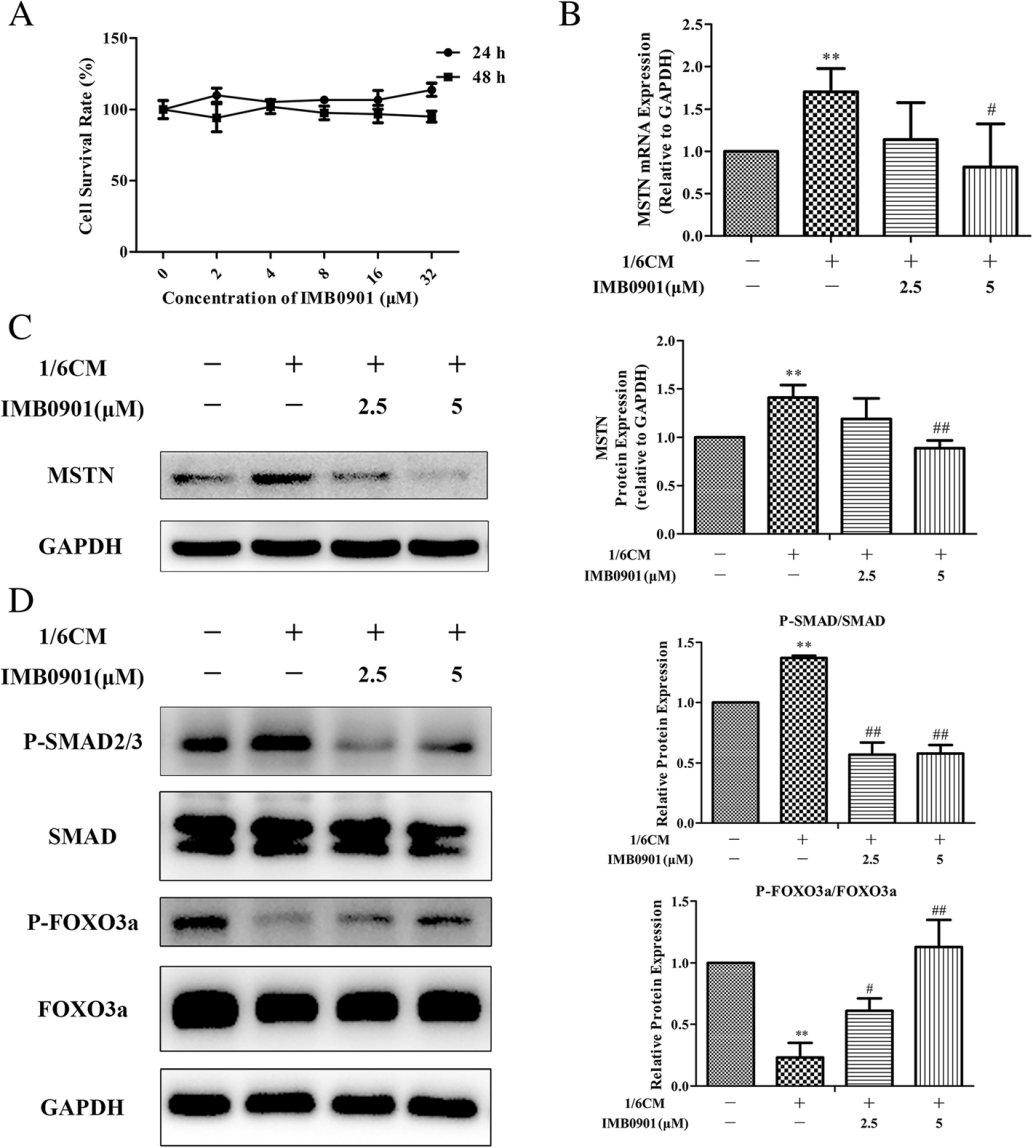
The effect of IMB0901 in the in vitro model. **a** The effect of IMB0901 on the survival rate of C2C12 cells. MTT assay was used to detect the survival rate of C2C12 cells after 24-h and 48-h treatment with IMB0901 at various concentrations. The values presented means ± SD from three experiments, each performed in triplicate. **b** The inhibition of IMB0901 on the MSTN mRNA expression. Real-time PCR was used to detect the effect of IMB0901 on the upregulation of MSTN during C2C12 myotube atrophy induced by C26 cell supernatant at mRNA level. There were four groups in this experiment: control (no CM or IMB0901), CM (1/6 CM), IMB0901 (1/6 CM + 2.5 μM IMB0901), and IMB0901 (1/6 CM + 5 μM IMB0901), for the following data. The data presented means ± SD from three experiments, each performed in triplicate. **P < 0.01 compared to the control group, ^#^*P* < 0.05 compared to the CM group. **c** The inhibition of IMB0901 on the MSTN protein expression. Western blotting was used to detect the effect of IMB0901 on the upregulation of MSTN during C2C12 myotube atrophy induced by C26 cell supernatant at protein level. The data presented means ± SD from three experiments. **P < 0.01 compared to the control group, ^##^*P* < 0.01 compared to the CM group. **d** The inhibition of IMB0901 on the MSTN signaling pathway in the in vitro cell model. Western blotting was applied to assess the effect of IMB0901 on the phosphorylation level of SMAD and FOXO3a during the process of C2C12 myotube atrophy induced by C26 cell supernatant. The values presented means ± SD from three experiments. **P < 0.01 compared to the control group, ^#^*P* < 0.05 and ^##^*P* < 0.01 compared to the CM group

To verify the effectiveness of IMB0901 on MSTN, we established the in vitro cell model that C2C12 myotube atrophy was induced by CM. There was no cytotoxicity of IMB0901 at less than 32 μM to C2C12 myoblasts using MTT assay (Fig. 2a). Due to no toxicity, 2.5 and 5 μM IMB0901 were chosen for the following experiments. Then, the effects of IMB0901 on the MSTN expression and signaling pathway were determined by real-time PCR and Western blotting. The results demonstrated that CM upregulated the MSTN mRNA (Fig. 2b) and protein (Fig. 2c) and there were significant differences (*P* < 0.01) between the control and CM groups. IMB0901 at 5 μM significantly downregulated the MSTN mRNA (Fig. 2b, *P* < 0.05) and protein (Fig. 2c, *P* < 0.01 vs. the CM group) induced by CM, and 2.5 μM IMB0901 exerted the similar effects on MSTN but there was no statistical significance between the two groups. Meanwhile, CM led to the increased P-SMAD and the reduced P-FOXO3a with a significant difference (Fig. 2d, *P* < 0.01 vs. the control group), and IMB0901 at a dose of 5 μM significantly inhibited the P-SMAD and increased the P-FOXO3a (Fig. 2d, *P* < 0.01 vs. the CM group).

### IMB0901 upregulated the expressions of myotube markers and protected the myotube morphology

**Fig. 3:**
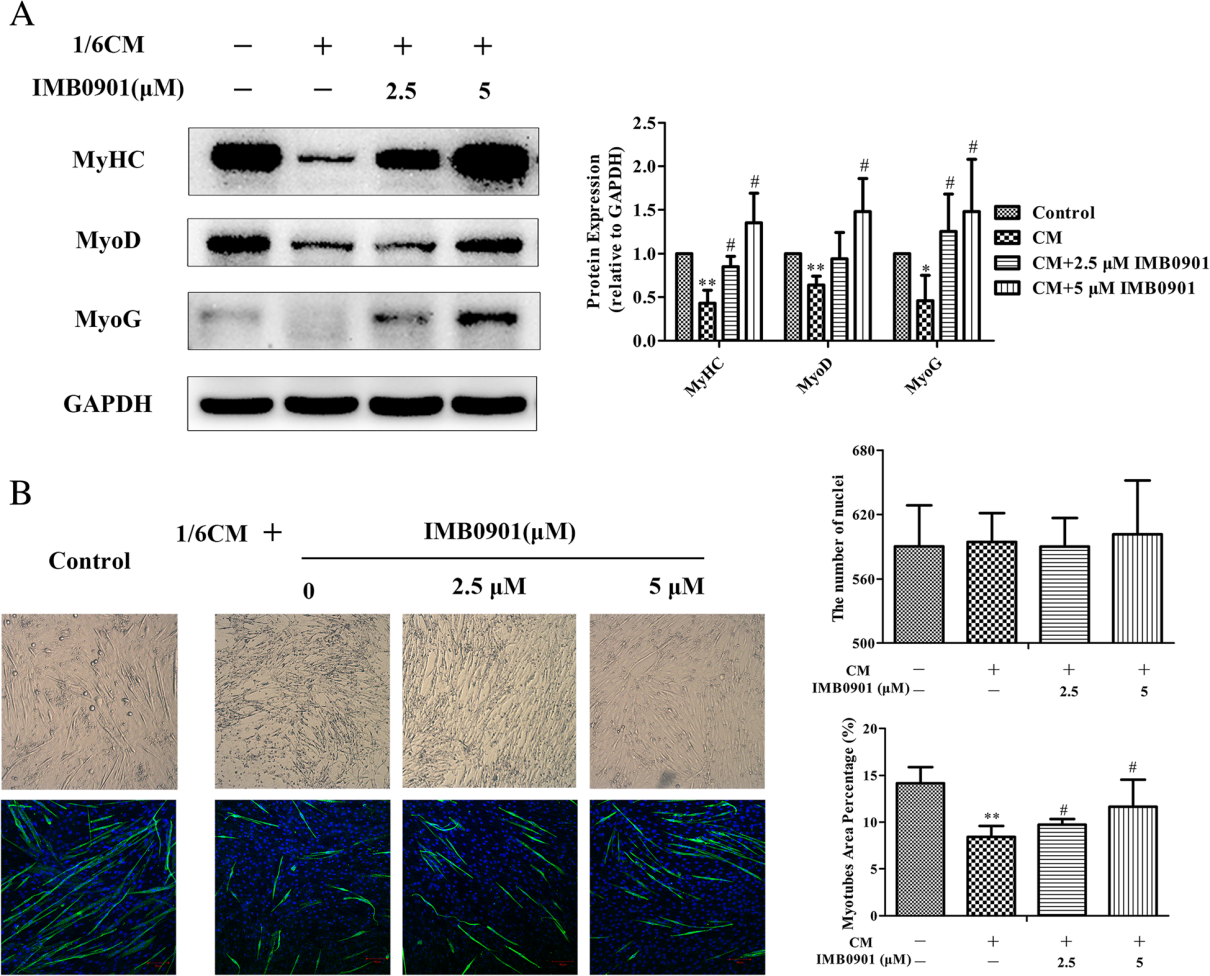
The therapeutic effect of IMB0901 in the in vitro model. **a** The effect of IMB0901 on the expression of three myotube protein markers MyHC, MyoD, and MyoG in the in vitro cell model. Western blotting was used to detect the effect of IMB0901 on the expression of three myotube protein markers MyHC, MyoD, and MyoG during the process of C2C12 myotube atrophy induced by C26 cell supernatant. **b** The effect of IMB0901 on the cytomorphology of C2C12 myotube atrophy induced by C26 cell supernatant. Left: The cell morphology during the process of C2C12 myotube atrophy induced by C26 cell supernatant was recorded with the inverted microscopy (upper), and the MyHC expression in C2C12 myotubes was detected by immunofluorescence and the nuclei was stained by DAPI (down). Right: The number of nuclei per field was examined by Image J software (upper), and the myotube area percentage was calculated by the immunofluorescence area divided by the horizon area (down). The values presented means ± SD from three experiments. *P < 0.05 and **P < 0.01 compared to the control group, ^#^*P* < 0.05 and ^##^*P* < 0.01 compared to the CM group

To determine the effect of IMB0901 on C2C12 myotube atrophy induced by CM in vitro, C2C12 myoblasts were exposed to 2% HS or IMB0901-treated/IMB0901-untreated 2% HS containing 1/6 CM. The change of three common myotube markers, myosin heavy chain (MyHC), myogenic differentiation 1 (MyoD), and myogenin (MyoG), was detected by Western blotting. In Fig. 3a, CM significantly downregulated the expressions of MyHC (*P* < 0.01), MyoG (*P* < 0.01), and MyoD (*P* < 0.05) as compared to the control group. IMB0901 inhibited the negative effect of CM and upregulated the expressions of MyHC, MyoG, and MyoD. There were significant differences (*P* < 0.01) between the CM and IMB0901 (5 μM) groups. In optical microscope (Fig. 3b (Left-upper)), C2C12 myotubes influenced by CM containing 5 μM IMB0901 were thicker and longer than atrophic C2C12 myotubes exposed to CM without IMB0901, which was parallel with the anti-MyHC immunofluorescence images (Fig. 3b (Left-down)). The effect of IMB0901 was quantified through the myotube area percentage with MyHC^+^ staining in the horizon representing the degree of MyHC expression as shown in Fig. 3b (Right). The IMB0901-treated group showed an increase of myotube area percentage with respect to the IMB0901-untreated group with a significant difference (Fig. 3b (Right-down), *P* < 0.05), which excluded the influence of the number of cells by the quantification of the number of nuclei per field (Fig. 3b (Right-upper)).

### IMB0901 lowered the expressions of Atrogin-1, MuRF-1, and ubiquitin and promoted phosphorylation of AKT, mTOR, and P70S6K in atrophied C2C12 myotubes

**Fig. 4:**
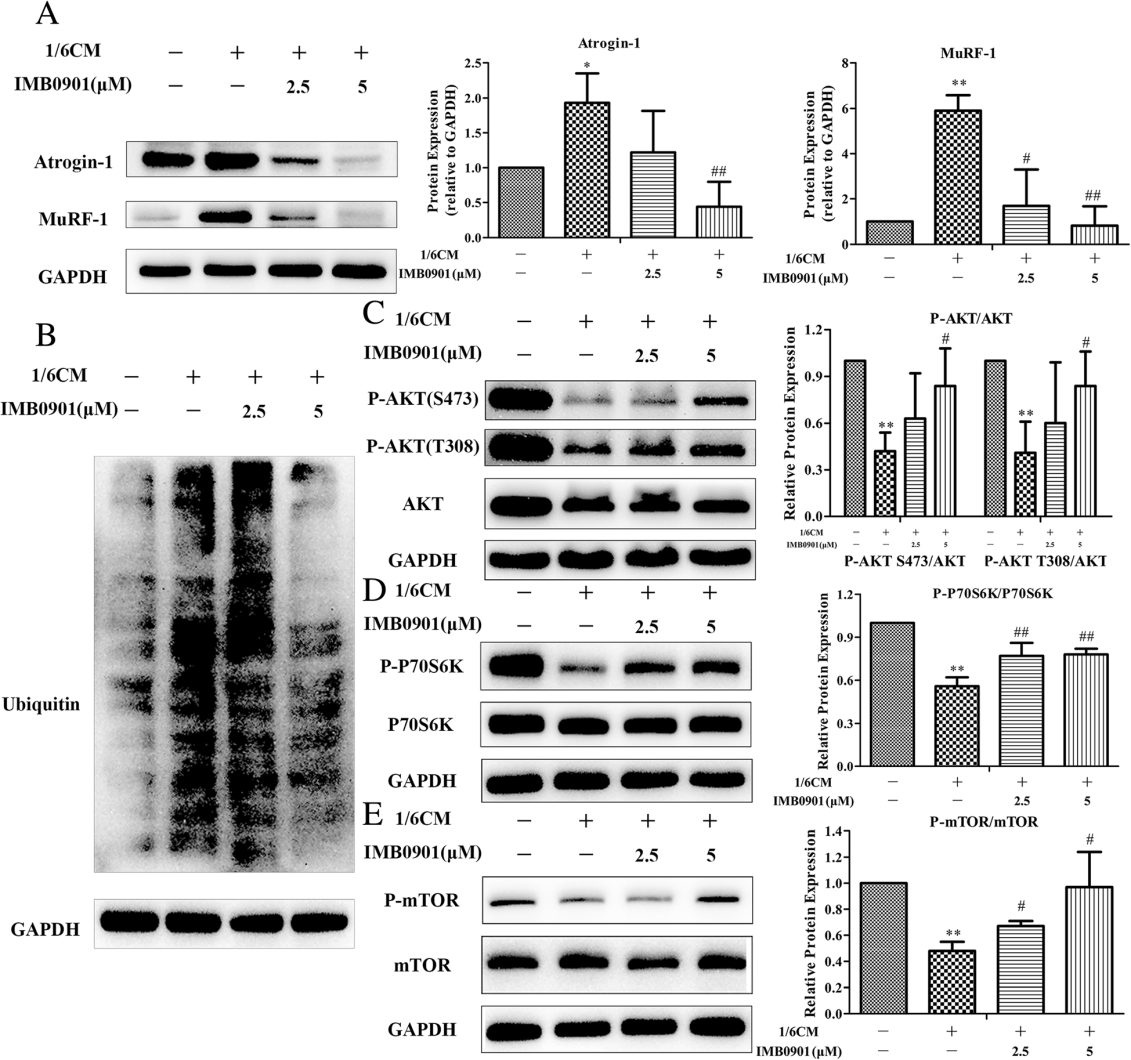
The mechanism of IMB0901 in the in vitro model. **a** The inhibitory effect of IMB0901 on two E3 ligases, Atrogin-1 and MuRF-1. Western blotting was used to detect the effect of IMB0901 on the expressions of two E3 ligases, Atrogin-1 and MuRF-1, during the process of C2C12 myotube atrophy induced by C26 cell supernatant. **b** The inhibitory effect of IMB0901 on the ubiquitin expression. Western blotting was used to detect the effect of IMB0901 on the ubiquitin expressions during the process of C2C12 myotube atrophy induced by C26 cell supernatant. **c**, **d**, **e** The positive effect of IMB0901 on phosphorylation level of AKT, P70S6K and mTOR in atrophied C2C12 myotubes. Western blotting was used to detect the phosphorylation of AKT, P70S6K and mTOR during the process of C2C12 myotube atrophy induced by C26 cell supernatant. The values were means ± SD from three independent experiments. *P < 0.05 and **P < 0.01 compared to the control group, ^#^*P* < 0.05 and ^##^*P* < 0.01 compared to the CM group

To clarify the influences of IMB0901 in the muscle metabolism network, we attempted to determine expressions of the two E3 ligases (Atrogin-1 and MuRF-1), ubiquitin, AKT, mTOR, and P70S6K in atrophied C2C12 myotubes. As shown in Fig. 4a–b, CM significantly increased the expressions of Atrogin-1 (Fig. 4a, *P* < 0.05), MuRF-1 (Fig. 4a, *P* < 0.01), and ubiquitin (Fig. 4b) in C2C12 myotubes as compared to the control group. At the same time, IMB0901 reduced the expressions of Atrogin-1 (Fig. 4a), MuRF-1 (Fig. 4a), and ubiquitin (Fig. 4b). There were significant differences (*P* < 0.01) between the CM and IMB0901 (5 μM) groups. Furthermore, it was shown that phosphorylation of AKT, mTOR, and P70S6K was significantly repressed by CM (Fig. 4c–e, *P* < 0.01 vs. the control group), which was reversed by 5 μM IMB0901. IMB0901 at a dose of 5 μM significantly elevated the ratios of P-AKT/AKT (Fig. 4c, *P* < 0.05), P-mTOR/mTOR (Fig. 4d, *P* < 0.05), and P-P70S6K/P70S6K (Fig. 4e, *P* < 0.01) as compared to the CM group.

### IMB0901 inhibited the weight loss of body, quadriceps, and gastrocnemius induced by C26 tumor, protect the cell morphology of quadriceps, and lowered the expressions of Atrogin-1 and MuRF-1 in BALB/c mice

**Fig. 5:**
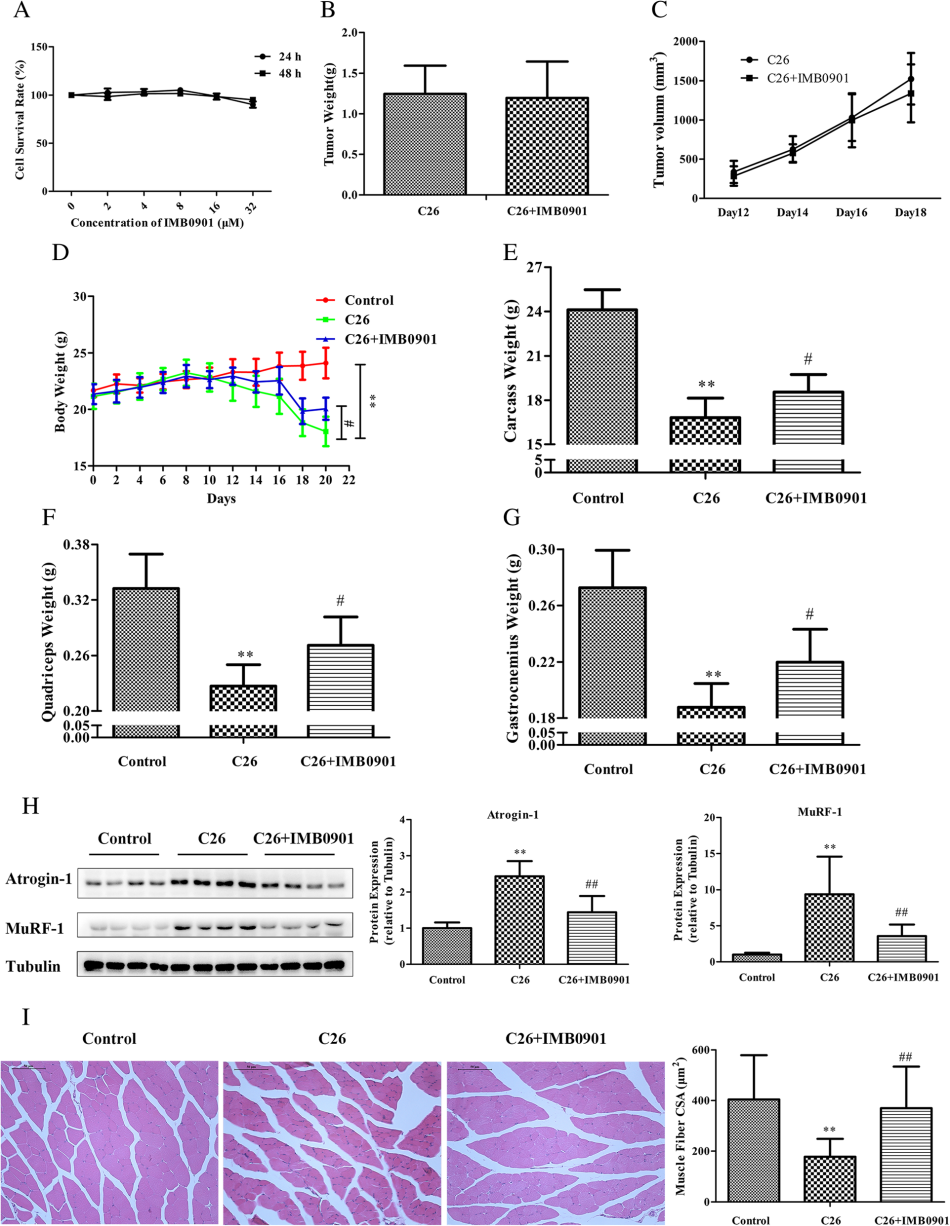
The therapeutic effect of IMB0901 in the C26 animal model. **a** The effect of IMB0901 on the survival rate of C2C12 cells. MTT assay was used to detect the survival rate of C2C12 cells after 24-h and 48-h treatment with IMB0901 at various concentrations. The values presented means ± SD from three experiments, each performed in triplicate. **b** The effect of IMB0901 on C26 tumor weights at endpoint. **c** The effect of IMB0901 on the tumor growth in in the group of C26 animal model and IMB0901 treatment. **d** The body weights of BALB/c mice at day 0~20. **e** The carcass weights of BALB/c mice. **f**, **g** The mass of quadriceps and gastrocnemius muscles in the C26 animal model. **h** The effect of IMB0901 on the expressions of two E3 ligases, Atrogin-1 and MuRF-1. Western blotting was used to detect the effect of IMB0901 on the expressions of two E3 ligases, Atrogin-1 and MuRF-1, in the quadriceps tissue. **i** Representative images of an H&E-stained cross-section of quadriceps from BALB/c mice and the average cross-sectional area (CSA) of the quadriceps fibers in the C26 animal model. There were three groups in this experiment: control, C26, C26 + IMB0901. The values presented means ± SD from 10 animals. **P < 0.01 compared to the control group, ^#^*P* < 0.05 and ^##^P < 0.01 compared to the C26 group

Firstly, the effect of IMB0901 on healthy mice was determined in vivo. However, it was surprising that IMB0901 had no effect on the body weight and the weights of quadriceps, gastrocnemius, WAT, and BAT when administered in healthy mice (Additional file 2: Table S1). The effects of IMB0901 on the C26 cells and tumors were detected in vitro and in vivo, respectively. Unfortunately, IMB0901 had no anti-tumor efficacy on the C26 cells at less than 32 μM, and C26 tumors were not affected by IMB0901 in a dose of 30 mg/kg (Fig. 5b, c).

To determine the therapeutic activity of IMB0901 on inhibiting muscle atrophy in vivo, the animal model was established that muscle atrophy in BALB/c mice was induced by C26 tumor. Compared with the control group, C26 tumor significantly decreased the body weight (*P* < 0.01), the carcass weight (*P* < 0.01), and the weights of quadriceps (*P* < 0.01) and gastrocnemius (*P* < 0.01) (Fig. 5d–g). However, IMB0901 significantly increased the body weight (*P* < 0.05), the carcass weight (*P* < 0.05), and the weights of quadriceps (*P* < 0.05) and gastrocnemius (*P* < 0.05) as compared to the C26 group (Fig. 5d–g). Furthermore, food intake and the weights of WAT and BAT were not affected by IMB0901 (data not shown).

The histological analysis of quadriceps tissue was performed. At first, the expressions of Atrogin-1 and MuRF-1 in quadriceps tissue were all determined by Western blotting. As was shown in Fig. 5h, the expressions of Atrogin-1 and MuRF-1 were significantly upregulated in the C26 group (*P* < 0.01 vs. the control group), which was reversed in the IMB0901 group with a significant difference (*P* < 0.01 vs. the C26 group). Histologically, the effect of IMB0901 was demonstrated by a significant increase in the fiber size of quadriceps tissue in the IMB0901 group as compared to the C26 group (Fig. 5i, *P* < 0.01). There exhibited a significant increase in average cross-sectional area (CSA) of the quadriceps fibers (Fig. 5i, *P* < 0.01) between the C26 and IMB0901 groups.

### IMB0901 did not affect the antitumor effect of doxorubicin

**Fig. 6:**
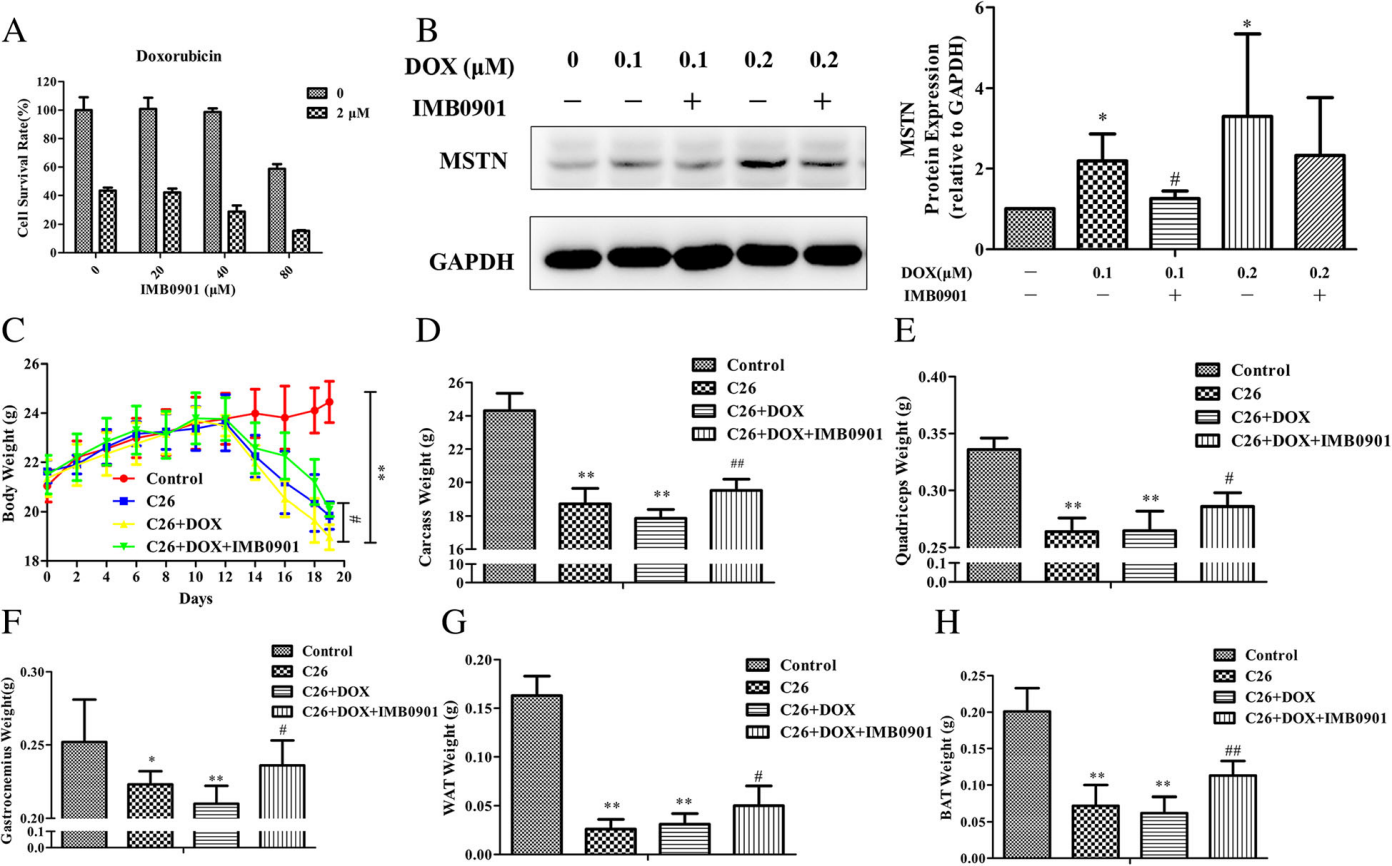
The effect of IMB0901 on the antitumor effect of doxorubicin in vitro and in vivo. **a** The effect of IMB0901 combined with doxorubicin on the cell survival rate of C26 cells. MTT assay was used to detect the cell survival rate of C26 cells after 24 h treatment with IMB0901 at various concentrations combined with doxorubicin. The values presented the mean ± SD from three experiments, each performed in triplicate. **b** The effect of IMB0901 on the MSTN expression change induced by doxorubicin in C2C12 myotubes. Western blotting was used to detect the expression of MSTN after 24-h treatment with IMB0901 and doxorubicin in C2C12 myotubes. The values were means ± SD from three experiments. *P < 0.05 compared to the control group, ^#^*P* < 0.05 compared to the DOX group. **c** The body weights of BALB/c mice at day 0~20. Doxorubicin was administered twice with 0.2 ml each at a dose of 10 mg/kg via the intraperitoneal injection. **d** The carcass weights of BALB/c mice. **e**–**h** The mass of quadriceps, gastrocnemius muscles, WAT, and BAT in the C26 and doxorubicin model. There were four groups in this experiment: control, C26, C26 + DOX, and C26 + DOX + IMB0901. The data were means ± SD from 10 animals. *P < 0.05 and **P < 0.01 compared to the control group, ^#^*P* < 0.05 and ^##^*P* < 0.01 compared to the C26 + DOX group

The above results suggested that IMB0901 could relieve the symptoms of cancer cachexia. Nevertheless, chemotherapy is inevitable in the clinic, so the in vitro effects of IMB0901 on the anti-tumor effects of common chemotherapy drugs were evaluated. We tested the effect of IMB0901 combined with cisplatin, doxorubicin, or gemcitabine in vitro on the cell survival of C26 cells. In Fig. 6a and Additional file 3: Figure S2A-B, it was observed that the survival of C26 cells was inhibited dose-dependently by doxorubicin, cisplatin, or gemcitabine, but IMB0901 did not change the anti-proliferation effect of the anti-cancer drugs.

The effect of IMB0901 on MSTN expression level altered by doxorubicin in C2C12 myotubes was detected. Doxorubicin upregulated the MSTN expression at concentrations of 0.1 and 0.2 μM (*P* < 0.05 vs. the control group), which was significantly inhibited by IMB0901 at a dose of 10 μM (Fig. 6b), and there was a significant difference (*P* < 0.05) between the 0.1 μM DOX and 0.1 μM DOX + IMB0901 group.

In the animal model, the effect of IMB0901 on the antitumor effect of doxorubicin was examined (Fig. 6c–h). The body weight, the carcass weight, and the weights of quadriceps, gastrocnemius, WAT, and BAT decreased significantly in the C26 group as compared with the control group (*P* < 0.01). In the C26 + DOX group, most of the above weights were lower than those in the C26 group. The body weight (*P* < 0.05), the carcass weight (*P* < 0.01), and the weights of quadriceps (*P* < 0.05), gastrocnemius (*P* < 0.05), WAT (*P* < 0.05), and BAT (*P* < 0.01) in the C26 + DOX + IMB0901 group were significantly higher than the C26 + DOX group.

## Discussion

Previous studies have shown that muscle atrophy is more important than fat loss in the development of cancer cachexia [1, 25, 26]. Ubiquitin-mediated proteolysis activated by MSTN is one of the mechanisms by which cancer cachexia leads to muscle atrophy [27]. The relationship between MSTN and cancer has not been fully elucidated, and there is even much controversy. However, it has been confirmed that the inhibition of MSTN can inhibit muscle atrophy induced by cancer cachexia [20, 28]. MSTN inhibitors in the preclinical or clinical trial stage are protein or gene drugs which may bring difficulties to the development and application because of the higher expense [22]. Therefore, MSTN was selected as a therapeutic target in our experimental system.

Based on the MSTN promoter activity or the MSTN signaling pathway, two stably transfected model cell lines including HEK293T-MSTNP or HEK293-SBE were successfully obtained and used to screen the molecule library, by which IMB0901 was selected from 26,000 molecules. IMB0901 possessed an inhibitory activity on MSTN signaling pathway similar to SB431542; thus, it was worth further investigation. Cash et al. [23] established the high-throughput drug screening model based on SBE in the MSTN signaling pathway. However, no high-throughput drug screening was reported based on the MSTN promoter activity. In this study, IMB0901 was screened by two methods. Among the 26,000 molecules, IMB0901 was the only one which has inhibitory effect on both the MSTN promoter activity and the MSTN signaling pathway. The other screened molecules exerted inhibitory effect on either the MSTN promoter activity or the MSTN signaling pathway. And we have not determined their therapeutic effects in vitro and in vivo. Combining the two methods for screening greatly reduced the probability of acquiring candidate compounds, but the obtained candidate compound exerted higher efficiency in subsequent drug target validation and pharmacodynamic studies. This method was worth further screening the molecular library in order to obtain more candidate compounds. Allen and Unterman [29] and Budasz et al. [30] reported that MSTN can upregulate its own expression as a cytokine. The HEK293 cell line expresses its receptors on the cell surface, so MSTN can upregulate the activity of its own promoter. As expected, IMB0901 suppressed this positive feedback mechanism of MSTN. Allen and Unterman [29] and Ma et al. [31] conducted an in-depth sequence analysis of MSTN promoter and found that there was an SMAD binding sequence. Therefore, the inhibitory effect of IMB0901 on the MSTN promoter activity might be related to the positive feedback regulation of SMAD and the whole MSTN signal pathway, and its specific mechanism was worth further exploration.

One of the most important advantages of MSTN as a therapeutic target is its regulation of both protein degradation and protein synthesis. In this process, AKT is a key point, and FOXO3a is the hinge linking the MSTN signaling pathway and ubiquitin-mediated proteolysis. FOXO3a is a transcriptional regulator of two muscle-specific E3 ligases, Atrogin-1 and MuRF-1. P-FOXO3a is regulated by AKT, and dephosphorylated FOXO3a is an active form. When FOXO3a is activated, more FOXO3a enters the nucleus and acts as a transcription factor to upregulate the expressions of Atrogin-1 and MuRF-1 [32]. Our results showed that C26 cell supernatant promoted the phosphorylation of SMAD and inhibited the phosphorylation of FOXO3a, while IMB0901 inhibited the upregulation of P-SMAD and reversed the downregulation of P-FOXO3a. Besides, IMB0901 suppressed the upregulated MSTN induced by C26 cell supernatant. Thus, the effectiveness of IMB0901 in the in vitro cell model was validated.

We validated that IMB0901 decreased the MSTN expression and inhibited the MSTN signaling pathway in the in vitro model. Subsequently, the efficacy of IMB0901 in the in vitro and in vivo models was tested. IMB0901 alleviated the C2C12 myotube atrophy induced by the C26 cell supernatant. Compared with the existing inhibitors of MSTN signaling pathway [28], IMB0901 was a small molecule inhibitor of MSTN. MSTN acts as a cytokine secreted by muscle and plays an important role in the interaction between muscle and fat [33], but its mechanism of action is not sufficient. IMB0901 could inhibit muscle atrophy but not the weight loss of adipose tissue in C26 mice model. However, IMB0901 could inhibit not only muscle atrophy but also the weight loss of adipose tissue in the C26 + DOX animal model. We supposed that this protective effect might be related to MSTN. The inhibition of ubiquitin-mediated proteolysis was the main mechanism of IMB0901, and in the in vitro model, IMB0901 simultaneously promoted protein synthesis, which was associated with the MSTN signaling pathway. Whether IMB0901 promote protein synthesis in C26 mice model still needs further experimental validation.

In the clinical treatment of cancer, chemotherapy is a routine method, and IMB0901 did not affect the inhibitory effect of cisplatin, doxorubicin, and gemcitabine on the growth of tumor cells in vitro. It has been reported that doxorubicin can aggravate muscle atrophy caused by cancer cachexia and the mechanism is also related to MSTN [34–36]. In this study, we found that IMB0901 had inhibitory effects on doxorubicin-induced MSTN upregulation in C2C12 myotubes and a significant alleviation of symptoms induced by C26 tumors and doxorubicin. The relationship between IMB0901 and doxorubicin is worth our further study.

Despite the fact that therapeutic effects were revealed by IMB0901, it also has limitations and extensive research needs to be performed on whether IMB0901 can be used to treat muscle atrophy caused by cancer cachexia. For example, IMB0901 was administered prior to tumor formation. The therapeutic effects of IMB0901 on animal models need to be validated with more animal models of cancer cachexia-induced muscle atrophy, such as the LLC model and the B16 model. In addition, we cannot detect IL-6 in the serum of mice, consistent with what has been reported by Seto [37]. IL-6 is only increased significantly at a later time. It is likely that we have terminated the experiment before the IL-6 can be measured.

It is disappointing that LY2495655 failed to confer clinical benefit in pancreatic cancer. Currently, myostatin inhibition has not been applied to clinical therapy of cancer cachexia. In the clinical trial, Golan et al. [38] report that pancreatic cancer patients cannot benefit from the treatment of the application of LY2495655, the anti-myostatin antibody. However, the study of myostatin should not been completely stopped for LY2495655. At first, it does not rule out that LY2495655 can be used in the treatment of tumors other than pancreatic cancer. Moreover, antibodies have different mechanisms for the treatment of tumors with small molecular inhibitors, which generally functions by blocking signaling pathways and the nonspecific killing of FC fragment, while small molecule of myostatin inhibitors may work by inhibiting myostatin expression and signaling pathways, and the potency of myostatin pathway inhibition is not the same as that of antibodies, so we study that small molecular inhibitors may produce different effects from LY2495655. In addition, a mouse anti-myostatin antibody increases muscle mass and improves muscle strength and contractility in the mdx mouse model of Duchenne muscular dystrophy, and its humanized equivalent, domagrozumab (PF-06252616), increases muscle volume in cynomolgus monkeys [39]. Our findings have identified IMB0901 as a lead compound targeting MSTN, which provided new insight into development of small-molecule MSTN inhibitor.

**Fig. 7:**
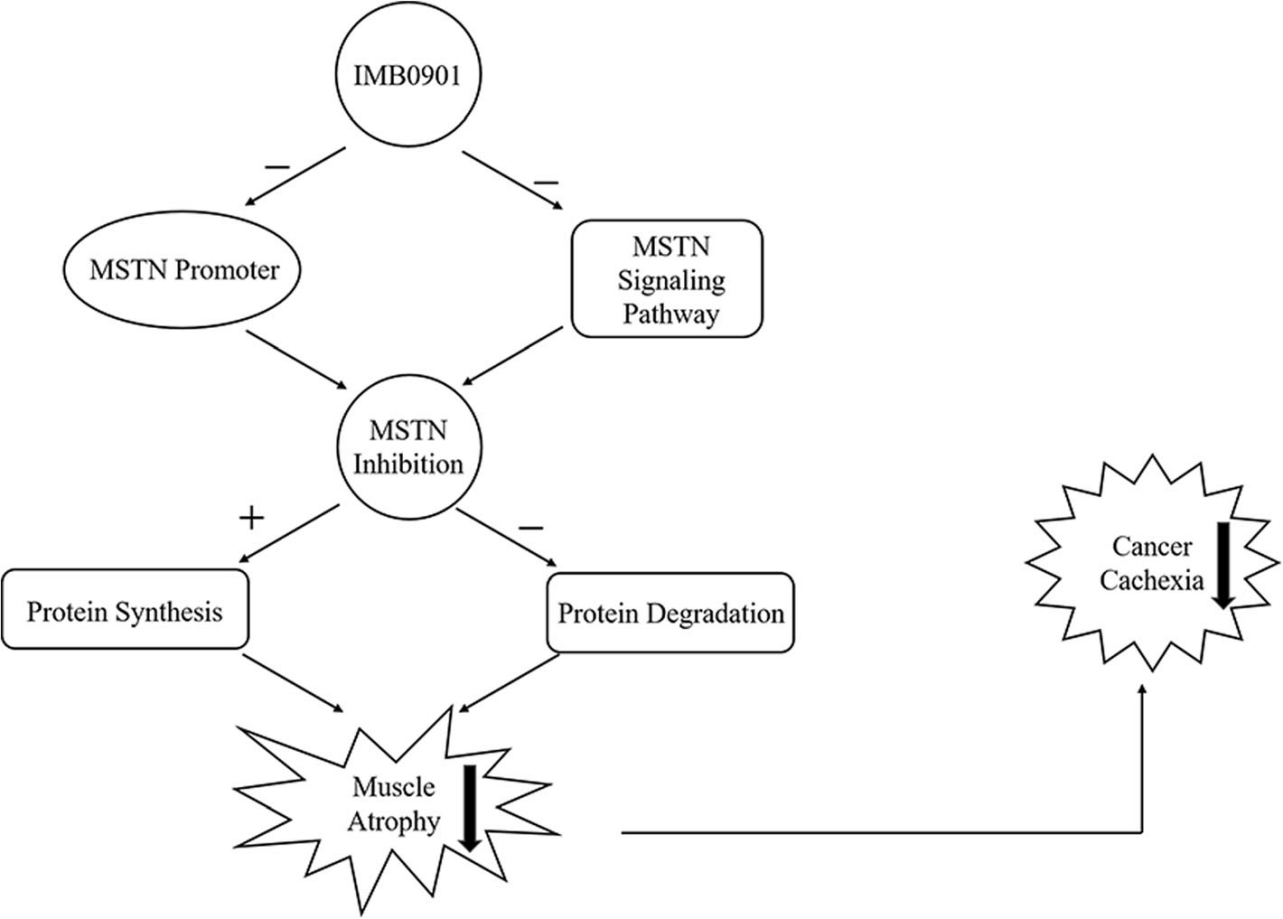
The mechanism schematic presentation of IMB0901 in inhibiting muscle atrophy induced by cancer cachexia

In summary, we tentatively clarify that IMB0901 is a dual MSTN inhibitor simultaneously inhibiting the MSTN promoter activity and the MSTN signaling pathway, thereby protecting muscle atrophy induced by cancer cachexia by inhibiting ubiquitin-mediated proteolysis and promoting protein synthesis (Fig. 7). IMB0901 also reduces some of the side effects of doxorubicin. In this study, IMB0901 is screened from a molecular library. This compound has a completely new therapeutic effect of alleviating cancer cachexia and inhibiting muscle atrophy both in vitro and in vivo. The mechanism of IMB0901 is initially discussed, and it is innovative and novel. This study provides theoretical and experimental bases for the development of IMB0901 as an attractive therapeutic option for the treatment of muscle atrophy induced by cancer cachexia. It also provides new research ideas for studying muscle atrophy induced by other chronic diseases.

## Conclusion

As a dual MSTN inhibitor, IMB0901 could alleviate cancer cachexia by inhibiting muscle atrophy, via the reduction of ubiquitin-mediated proteolysis and the elevation of protein synthesis. These findings collectively suggest that IMB0901 is a promising leading compound for the management of muscle atrophy induced by cancer cachexia.

## Additional files

Additional file 1: **Figure S1.** The construction flowchart of the recombinant plasmid with luciferase reporter gene based on the MSTN promoter activity. (TIF 239 kb)

Additional file 2: **Table S1.** The effect of IMB0901 on the weights of body, quadriceps, gastrocnemius, WAT, and BAT when administered in healthy mice. (DOCX 16 kb)

Additional file 3: **Figure S2.** The effect of IMB0901 combined with cisplatin (A) or gemcitabine (B) on the cell survival rate of C26 cells. MTT assay was used to detect the cell survival rate of C26 cells after 24 h treatment with IMB0901 at various concentrations combined with cisplatin or gemcitabine. (TIF 1420 kb)

## Abbreviations

ActRIIB: Type II B receptors
C26: Colon-26 adenocarcinoma
CM: C26-conditioned medium
DM: Differentiation medium
GM: Growth medium
HS: Horse serum
IGF1: Insulin growth factor 1
MSTN: Myostatin
MyHC: Myosin heavy chain
MyoD: Myogenic differentiation 1
MyoG: Myogenin
TGFβ: Transforming growth factor β
