# Supplementary figures and images for: IMB0901 inhibits muscle atrophy induced by cancer cachexia through MSTN signaling pathway
Source: Skelet Muscle. 2019 Mar 28;9:8. doi: 10.1186/s13395-019-0193-2 (PMC6437903; doi:10.1186/s13395-019-0193-2)

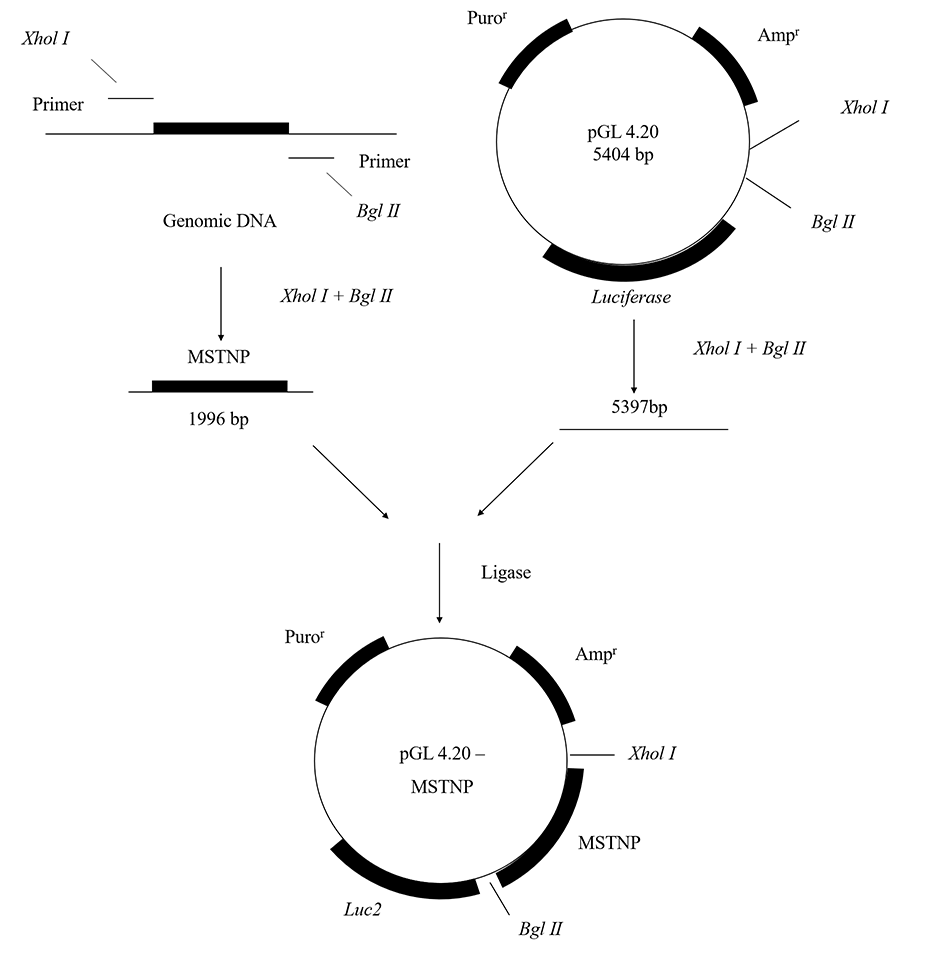

Supplement: Supplementary file 1 — Figure S1. The construction flowchart of the recombinant plasmid with luciferase reporter gene based on the MSTN promoter activity. (TIF 239 kb) [file 13395_2019_193_MOESM1_ESM.tif]

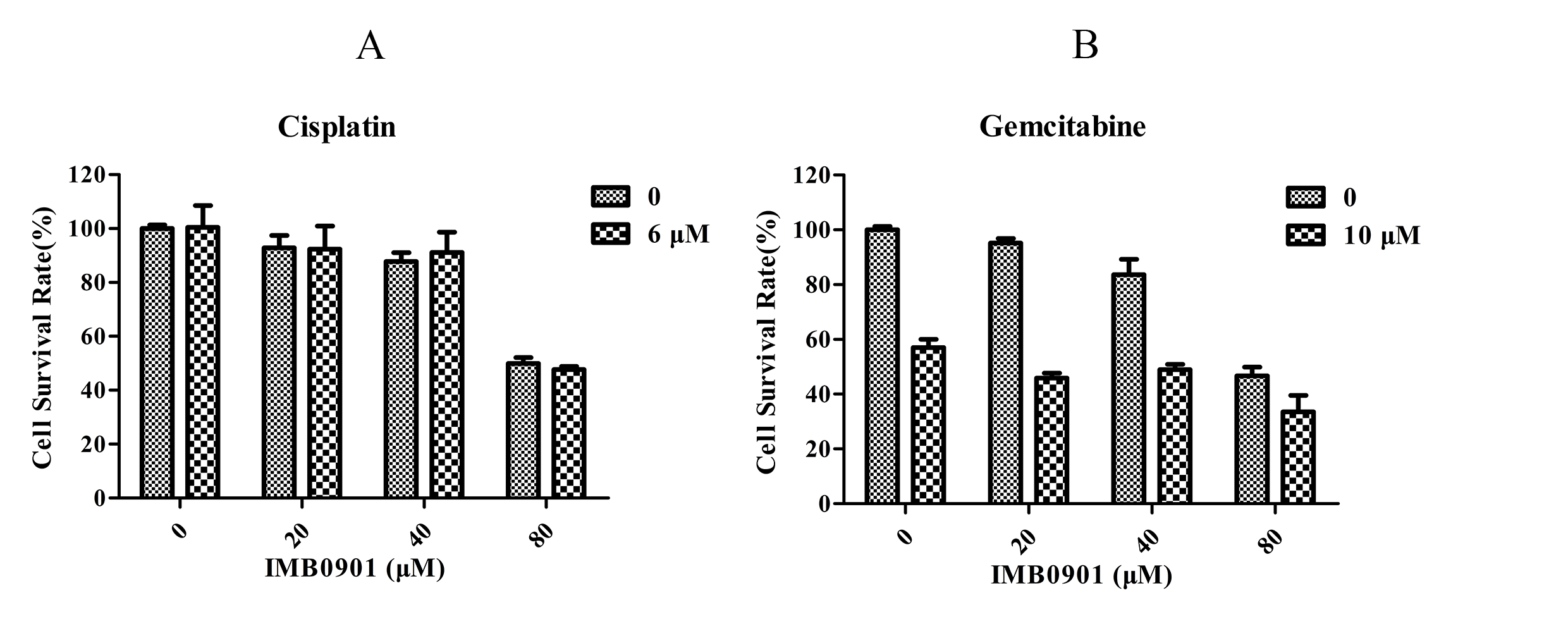

Supplement: Supplementary file 3 — Figure S2. The effect of IMB0901 combined with cisplatin (A) or gemcitabine (B) on the cell survival rate of C26 cells. MTT assay was used to detect the cell survival rate of C26 cells after 24 h treatment with IMB0901 at various concentrations combined with cisplatin or gemcitabine. (TIF 1420 kb) [file 13395_2019_193_MOESM3_ESM.tif]
