# Supplementary material for: IMB0901 inhibits muscle atrophy induced by cancer cachexia through MSTN signaling pathway
Source: Skelet Muscle. 2019 Mar 28;9:8. doi: 10.1186/s13395-019-0193-2 (PMC6437903; doi:10.1186/s13395-019-0193-2)
Supplement: Supplementary file 2 — Table S1. The effect of IMB0901 on the weights of body, quadriceps, gastrocnemius, WAT, and BAT when administered in healthy mice. (DOCX 16 kb) [file 13395_2019_193_MOESM2_ESM.docx]

**Table S1** The effect of IMB0901 on the weights of body, quadriceps, gastrocnemius, WAT, and BAT when administered in healthy mice.

| Group | Body(g) | Quadriceps(g) | Gastrocnemius(g) | BAT(g) | WAT(g) |
| --- | --- | --- | --- | --- | --- |
| Control | 24.26±1.29 | 0.175±0.017 | 0.128±0.011 | 0.158±0.028 | 0.118±0.026 |
| IMB0901 | 23.60±1.79 | 0.164±0.016 | 0.121±0.015 | 0.155±0.024 | 0.132±0.018 |

Note: The weight of quadriceps and gastrocnemius was from one leg in mice different from Fig.5.
